# From scenario to mounting risks: COVID-19’s perils for development and supply security in the Sahel

**DOI:** 10.1007/s10668-022-02303-9

**Published:** 2022-04-07

**Authors:** Mohammad Al-Saidi, Suhair A. Gayoum Saad, Nadir Ahmed Elagib

**Affiliations:** 1 Policy, Planning and Development Program, Center for Sustainable Development & Department of International Affairs, College of Arts and Science, Qatar University, Doha, Qatar; 2 Al Rawabi Dairy Company, P.O. Box 50368, Dubai, United Arab Emirates; 3 Institute of Geography, Faculty of Mathematics and Natural Sciences, University of Cologne, Albertus-Magnus-Platz, 50923 Cologne, Germany

**Keywords:** COVID-19, Risk, Security, Sustainable development, International aid, Sahel

## Abstract

The African Sahel countries are inherently fragile, environmentally insecure and economically weak. This paper underscores the compounded impacts brought about by the COVID-19 pandemic on resource supply security and, hence, the long-term development of the region. It outlines the Sahel-specific COVID-19 scenario by firstly highlighting the underlying vulnerabilities and later linking the health sector outcomes to increased political instability and environmental insecurity, particularly the deterioration of food security. In this sense, this paper shows from a region-wide perspective how COVID-19 in the Sahel is associated with enlarged sociopolitical developmental perils. Lower remittance sent by expatriates, violent conflicts, increased cross-border terrorism and migration, discriminant mobility restrictions of people and goods, weak national healthcare infrastructures, bottlenecks in international aid, pressures on the education system and recent climate extremes are some revealing examples of aggravators of the impacts on the supply of vital resources, such as food. This paper also shows the importance of considering the close interlinks between health, food and political stability in the Sahel. There is a paramount need for more comprehensive approaches linking human health to other sectors, and for re-considering local sustainable agriculture. To avoid prolonged or recurrent humanitarian crises, the Sahel countries need to strengthen response capacities through public sector-led responses. Examples of these responses include reinforced national disaster programs for the vulnerable, support to sustainable agriculture and food markets, improved performance and communication of public sector relief, state-based cooperation, building of regional alliances and peacemaking efforts.

## Introduction

**Fig. 1:**
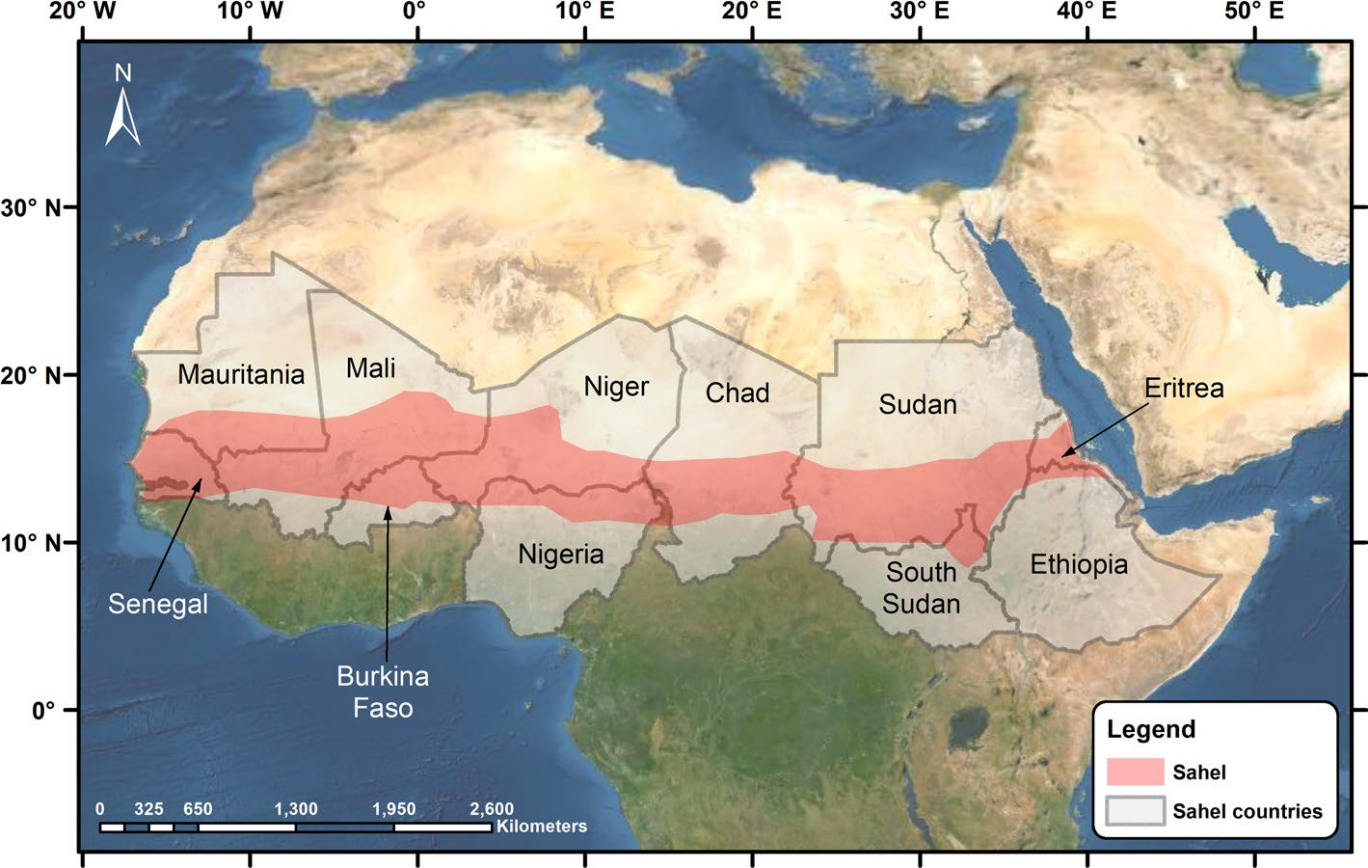
Map of the Sahel region and countries

For a long time, the Sahel countries (Fig. 1) have faced a combination of security, environmental and developmental problems. These problems have placed the region among the poorest in the world. The current COVID-19 crisis has been described as a game changer for development in Africa although COVID-19’s repercussions are still unfolding (Barbier & Burgess, 2020; Brown, 2021; Sachs et al., 2020).

COVID-19 arrived in the Sahel region at the end of February 2020 after one month of announcing it as pandemic. The first case announced in the region was in Nigeria on February 28, 2020, then Senegal, till reaching the whole Sahel by March 26, 2020 in Mali. Until the third week of October 2021, the confirmed COVID-19 cases in the Sahel exceeded 700 thousands, compared to 6 million in the whole Africa, with an average mortality rate of 2% which is similar to the global average rate (WHO, 2021a). It is likely that the real number is far above these official statistics. Meanwhile, donor organizations have been sounding the alarm about the impact of the COVID-19 crisis while few academic studies have highlighted the compounded health and economic impacts in Africa (e.g., Obayelu et al., 2021; Elebesunu et al. 2021). Although the number of COVID-19 cases in the Sahel region has been lower than in other regions, it imposed hard pressure on health systems (Molyneux et al., 2020; Dasgupta & Robinson, 2021), with impacts extending to other areas such as the food sector. This situation coupled with economic shrinkage after partial or complete lockdown could reflect desperately on the ongoing efforts to eliminate food insecurity and other health problems in the region, especially malnutrition (UNICEF, 2020b), malaria (WHO, 2019), tuberculosis (Okeke, 2019) and children routine immunization programs (Mihigo et al., 2015). International organizations also started to face funding problems if donating countries reduce or stop aid (United Nations 2020b). These common impacts on the African region necessitate a much broader analysis beyond single disturbance. For the Sahel, studies linking COVID-19 to common regional challenges and developmental repercussions are lacking.

During the COVID-19 pandemic, there were several calls to increase the preparedness and ramp-up responses of Africa, and specifically in vulnerable regions such as the Sahel. The suggested measures targeted the health sector through testing and containment (Senghore et al., 2020), the food sector through sustainable local agriculture (Hickey & Unwin, 2020), improved access to water (Anim & Ofori-Asenso, 2020), wastewater surveillance and monitoring (Street et al., 2020), resilience in the tourism sector (Rogerson & Baum, 2020) or aid for the education sector (Lewin, 2020). Almost two years into the crisis, there is a lack of systematic analyses of the success of response efforts and the compounded effects of COVID-19 on the Sahel region.

This paper focuses on the impacts on a vital supply sector, namely food, and relates these impacts to the developmental context of the region. It illustrates a unique Sahel scenario, namely how the COVID-19 pandemic has aggravated underlying vulnerabilities in areas of health, environmental and political stability, and thus increased sociopolitical risks and worsened supply insecurities. In fact, considering the youthful population of Africa, COVID-19 might result in a lower rate of mortality and morbidity than in other places, or in “manageable” effects on individual sectors due to previous experiences with disease outbreaks (Winning, 2020). COVID-19 might, nonetheless, prove to be a more serious stressor for the overall economic, developmental and security context of the Sahel than in some other regions with higher COVID-19 cases. Due to the specific context of the Sahel, which exhibits a range of long-standing vulnerabilities, the region requires more specific and coherent analyses. The multifaceted turbulences caused by the COVID-19 have been evident over the course of the pandemic. They can change both priorities and perceptions of international cooperation and development (Oldekop et al., 2020). The socioeconomic and political impacts may last for years if not decades.

**Fig. 2:**
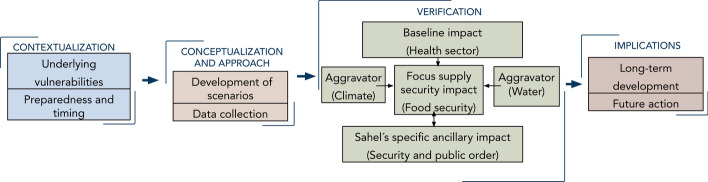
Research outline and steps

Figure 2 outlines the overall design and research steps of this paper. We aim at providing a structured and aggregated analysis of the COVID-19 impacts on the Sahel region by considering its specific development and environmental context. This analysis can help break up the storm brought about by the COVID-19 through understanding the broader picture of the Sahel developmental context and addressing weak spots. The paper starts by describing the Sahel’s recent context and, then, outlines the specific Sahel scenario in facing the COVID-19 storm of health sector disruptions compounded by interaction with environmental and sociopolitical aggravators. At the same time, we focus in the verification of the Sahel scenario on the compounded impacts on the food sector. Using country-level examples and conceptualizations of the COVID-19 scenarios, the Sahel case is embedded in the specific reality and the recent vulnerabilities of this developing region, particularly the factors related to aid dependence, political fragility, supply insecurity and susceptibility to climatic-related disasters and extremes. The example of food security illustrates how those underlying vulnerabilities, together with the pandemic impacts, created a perfect storm of developmental perils. In discussing the implications of this Sahel scenario, we emphasize the need for specific frameworks and tailored strategies for long-term resilience in the Sahel.

## The Sahel’s COVID-19 case: caught in a storm

### Recent developmental context: a bad timing for COVID-19

**Fig. 3:**
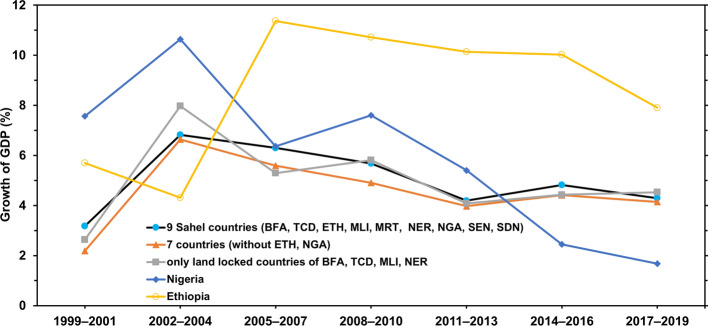
Average growth of Gross Domestic Product (GDP) in the Sahel region 1999–2019 at market prices using constant local currency. Abbreviations: BFA: Burkina Faso; TCD: Chad; ETH: Ethiopia; ERI: Eritrea; MLI: Mali; MRT: Mauritania; NER: Niger; NGA: Nigeria; SEN: Senegal; SDN: Sudan. Source: The World Bank (2020) for data from 1999–2001; African Development Bank Group (2020) for GDP estimations for the year 2019. Eritrea is not considered due to lack of consistent data

According to the World Bank (2020), the last couple of years did not entail many upward signs in development indicators. Economic growth has been generally lower in the last decade, averaging 4.9% for all the nine Sahel countries, except Eritrea, in 2009–2018 in comparison with 5.8% in 1999–2008. There has been a slight improvement in 2019 when economic growth grew to an average of around 5% compared with only an average of around 4% in three years before (African Development Bank Group, 2020; World Bank, 2020). Except for the strong performance of Ethiopia, economic growth has been erratic (Fig. 3), especially true for the landlocked, resource-poor and politically fragile states of Burkina Faso, Chad, Niger and Mali. In addition, the humanitarian crisis has worsened in the Sahel in the last decade. Migration within the Sahel countries and toward the exterior increased due to environmental degradation and security challenges (OCHA, 2016). In seven Sahel countries (Burkina Faso, Mali, Mauritania, Niger, Sudan, Senegal and Chad), the number of internally displaced people due to conflicts and natural disaster has increased lately, with the total number in these countries jumping from around 448 thousand people in 2018 to 1.4 million in 2019 (IDMC, 2020). Most recent concerns tie the rise of Jihadist groups in the Sahel to crime and contraband (Barkindo, 2020). Shortly before the COVID-19 outbreak, scholars have been warning of a catastrophe in the Sahel. Rapidly growing population, climate extremes, food insecurities and violence meant that the Sahel was heading toward “a perfect storm” of misery (Graves et al., 2019). COVID-19 might have brought the storm into the heart of the Sahel.

In the Central Sahel (Burkina Faso, Mali and Niger), the number of food insecure people rose to 4.8 million in May 2020 in comparison with 3.9 million prior to COVID-19 (WFP, 2020). In July 2021, an increase to 8.7 million was reported (WFP, 2021). This increase was also linked to a severe lean season and massive locust swarms (Salih et al., 2020; WFP, 2020). The lockdowns and border restrictions imposed have led to livelihood losses in the agro-pastoral industries and severe consequences for food markets and supply chains (Refugees International, 2020). Considering the combined use of land for agriculture, fuelwood and water-related services, the impacts are probably well beyond the food sector (Elagib & Al-Saidi, 2020). In addition, the security threat has increased with some reports of terrorist groups exploiting the pandemic to destabilize governments (UN News, 2020b).

### Underlying vulnerabilities: little prepared for a pandemic

In order to face the current pandemic, the Sahel countries have responded with emergency plans while taking measures for ensuring basic supply. However, the responses require a level of preparedness and resource endowment, both of which have been lacking in the Sahel despite some country-level differences (Collier, 2007). In fact, all the Sahel countries rank very low on development and environmental indices, with relatively better scores for Ethiopia, Nigeria and partly Senegal (Table 1). All the Sahel countries are considered prone to climate/water-related disasters, such as droughts and floods (Elagib & Elhag, 2011; Elagib et al., 2021; Hulme, 2001; Kerr, 1985; Tarhule, 2005; Tschakert et al., 2010). They also rank rather low on the achievement of food and energy securities, and are among the world’s most fragile states (Parry et al., 1999; World Bank, 2013). These compound economic and environmental problems in the Sahel mean that certain communities depend on international aid (Somerville, 2018). Environmental vulnerability and aid politics led to the political marginalization of some ethnic groups and remote communities (Raleigh, 2010). In addition, the Sahel region exhibits strong migratory flows. Firstly, the pastoral and subsistence-based livelihoods in the Sahel mean that mobility and labor migration (across states) are high. Secondly, environmental degradation and climate change can increase migration in disaster-prone areas (Hummel, 2016). Thirdly, long-standing migration toward Europe has received more attention lately, leading to more controls, border restrictions and aid (Raineri & Rossi, 2017). Even without COVID-19, there have been massive pressures on Sahel governments to address insecurities and provide opportunities for their young and rapidly growing populations.

**Table 1:** Performance of the Sahel countries in relation to key global indices of health, development and environmental security

Global Index (year of the index version; total number of ranks)^a,b^	BFA	TCD	ETH	ERI	MLI	MRT	NER	NGA	SEN	SDN
GFSI: Global Food Security Index (2019; 113)	87	109	91	–	80	-	89	94	81	99
GHSI: Global Health Security Index (2019; 195)	145	150	84	178	147	157	132	96	95	163
GESI: Global Energy Security Index (2020; 229)	129	160	81	145	139	144	108	102	73	125
SFI: State Fragility Index (2020; 178)	142	172	158	161	163	146	160	165	107	171
HDI: Human Development Index (2019; 189)	182	187	173	182	184	161	189	158	166	168
WorldRiskReport (Focus: Water) (2019; 180)	147	150	116	99	145	114	159	157	137	122
SDGI: Sustainable development Goals Score Index, SDGI (2020; 166)	137	164	136	–	156	130	157	160	127	159

^a^The GFSI covers affordability, availability and quality of food: https://foodsecurityindex.eiu.com/. The GHSI assesses health security and capabilities across the categories of prevention, detection and reporting, rapid responses, health system, compliance with international norms and risk environment: https://www.ghsindex.org/. The GESI is a comprehensive energy index proposed by Azzuni & Breyer (2020), and includes several energy, environmental and political economic dimensions. The SFI measures the vulnerability to conflict or state collapse through the categories of cohesion (security apparatus, fractionalization and group grievances) as well as other socioeconomic and political categories: https://fragilestatesindex.org/. The HDI is a common United Nations developmental index using income, health and educational indicators: http://hdr.undp.org/. The WorldRiskReport 2019 measures the risk to disaster caused by extreme natural events with a focus on water-related disasters, including categories of risk, hazard, exposure, susceptibility, coping and adaptation: https://weltrisikobericht.de/. The SDGI tracks country performance on the 17 Sustainable development Goals (SDGs), as agreed by the international community in 2015 with equal weight to all 17 goals (Sachs et al., 2020)

^b^A higher rank indicates a worse scoring (e.g., 113 from 113 means the worst performing country). The ranking order in some indices was reversed, namely in the State Fragility Index (now, rank 178 = most fragile) and the WorldRiskReport (now rank 189 = highest risk)

*BFA* Burkina Faso; *TCD* Chad; *ETH* Ethiopia; *ERI* Eritrea; *MLI* Mali; *MRT* Mauritania; *NER* Niger; *NGA* Nigeria; *SEN* Senegal; *SDN* Sudan

The health systems in the Sahel region, similar to all African countries, are very fragile with challenges of poor workforce, financial problems and poor management practices (Oleribe et al., 2019). In Africa, the average density of healthcare workers per 10,000 people is 57 compared to 583 for higher income countries (WHO, 2018). During the pandemic, stressed health systems in many countries have increased deaths from other diseases or worsened communities’ health. For example, people are avoiding going to hospitals even after having sever health problems, such as heart attacks and normal chronic diseases (Hafner, 2020; Kaufman, 2020). Despite the weak health situation, some analysts predict that, due to its different demographic characteristics, the health impact of COVID-19 for the Sahel region could actually be lower than in other world regions. The share of population above the age of 50 is only 10% for sub-Saharan Africa compared to 40% in Europe (OECD, 2020a).

## Methods: assessing and outlining the COVID-19 scenarios

### Conceptual framework and scenario descriptions

**Fig. 4:**
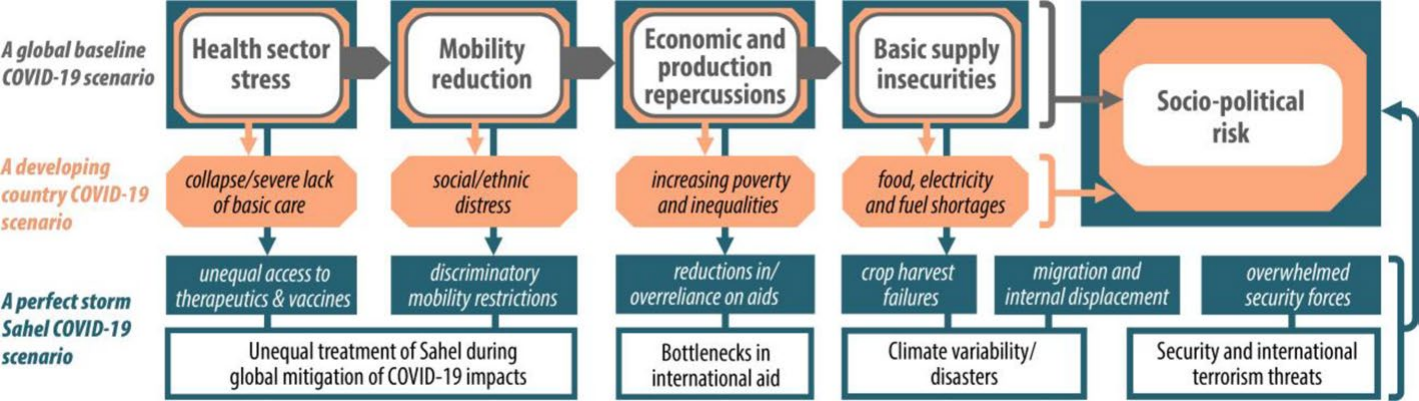
COVID-19 scenarios in the context of developing countries in general and the Sahel region in particular. The different frame/color combinations distinguish the scenarios of negative pressures caused by COVID-19 that can be enlarged due to additional aggravating factors

The high vulnerability of basic care, supply and security systems in the Sahel suggests serious impacts of COVID-19. The pandemic trajectory has been quite specific in the region, and is explained in this paper using a conceptual framework depicted in Fig. 4. This framework seeks to organize the analysis in this paper by providing some expected impacts and ancillary effects that we outline in three scenarios. The first scenario represents the basic or universal course of COVID-19 impacts. The second scenario is built by adding general ancillary effects (probable and additional events) specific for developing countries. The third scenario extends developing countries scenario by adding another layer of aggravating factors specific to the Sahel region. These three scenarios can lead to different levels of sociopolitical risks, i.e., tangible disruptions of functioning of societies in terms of socioeconomic order and political systems. The focus of this paper is on synthesizing current evidence on COVID-19 in the Sahel in order to explain and discuss the third scenario (Sects. 4 and 5). However, the three scenarios are introduced briefly here. First, the core or common scenario is valid worldwide where COVID-19 causes a stress on the health sector, leading to lockdowns, a slowdown of economic and production activities and, eventually, supply securities. This phase meant that the Sahel countries were imposing lockdowns and restrictions on economic activities. The Sahel countries have requested increased funding from multilateral donors, cancelled upcoming elections, introduced some national support funds and utilized existing health monitoring systems supported by the World Health Organization (WHO) during the Ebola outbreaks (Gandhi et al., 2020).

Second, developing countries might experience more severe disruptions. Under this scenario, the basic health care system can face collapse while social and ethnic distress, poverty, inequalities and shortages in basic supply are expected to increase. There is already some evidence of these effects in the Sahel such as the earlier-mentioned increase of food insecurity. In June 2020, the United Nations (UN) Secretary-General António Guterres warned of a dire humanitarian crisis caused by economic decline, shortages of food supply, lower remittances from workers abroad and security risks (Lederer, 2020). There have been some outcries against the economic hardships of the lockdown measures. The lockdown can undermine civil liberties and increase marginalization of groups in peripheral areas (Bisson et al., 2020).

Third, a longer-term scenario includes Sahel-specific aggravating factors. This scenario can lead to a perfect storm of humanitarian jeopardy. Some of these factors have already started to materialize. We already explained how climate variability and natural disasters, such as changes in rainfall and locust swarms, increased the humanitarian situation in the Sahel in recent years. Recently, the UN has asked for emergency funds to face the locust swarms in the Sahel during the COVID-19 crisis (Parkin, 2020; Salih et al., 2020). Furthermore, the security situation has been tense in the region with recent terrorist attacks and an escalation of the Libyan conflict that can affect the Sahel. Another unknown but important factor in the Sahel scenario is international aid. Aid agencies have called for increased funding for the Sahel, with the UN appealing for $2.8 billion to reach 17 million people in need, out of which only 18% were received as of May 2020 (Schlein, 2020). The European Union and other donors have pledged further aid. Some reports suggested a mobilization of $57 billion by official creditors (including IMF and the World Bank) and $13 billion by private creditors for Africa in 2020 (Modern Diplomacy, 2020). However, considering the increasing fiscal problems of states worldwide, it is not sure how much aid is going to reach the Sahel or be dedicated to the immediate COVID-19 impacts. Donors might decide to provide debt relief instead of additional aid or to direct more aid toward improving the access of Africa and the Sahel to COVID-19 vaccines. In fact, the access of Sahel countries to COVID-19 therapeutics and potential vaccines is a key factor in this crisis. The UN has advocated equal and quick access for the group of underdeveloped countries. Furthermore, during the course of the COVID-19 crisis, Sahel countries can face significant discriminations with regard to mobility of people and goods. For example, the conditioning of COVID-19 testing for international travel and the rise in air ticket and freight costs are bound to be more hurtful to low-income countries.

### Data collection and limitations

This paper uses the above-described framework to investigate the COVID-19 scenario in the Sahel and its repercussions on development and supply security in the context of the pre-crisis vulnerability of the region. Data collected for this analysis can be divided in two parts. First, to depict the early stage of the crisis, data were collected continuously upon the onset of COVID-19 crisis. Here, secondary data mainly from international organizations were used to depict the Sahel’s vulnerability (see Sect. 2). Besides, data on COVID-19’s early impacts were collected from secondary resources and the scarce academic literature during the period March 2020 until October 2021. Second, a systematic literature review was conducted in October 2021 to complement and validate the scenarios’ analysis. A Scopus research using the keywords “Sahel” and “COVID-19″ only results in 9 documents, indicating the scarce knowledge using a regional viewpoint. However, adding the names of the Sahel countries, more than 1400 entries are found in Scopus (stand mid October 2021), with 804 entries from medicine, and 287 in social sciences. Since this paper focuses on aspects relating to development, supply security and sociopolitical risks, we limited the literature selection by seeking studies (between 2020 until October 2021) with the following keyterms in the title, abstract or keywords: COVID-19, the Sahel or any Sahel country name, as well as any of the terms "security,” “supply,” “development,” “food,” “risk.” The resulting dataset of 572 that included a large portion of social sciences studies (174 papers) was sorted out by including only original research or review papers as well as excluding papers with a too-specific focus (e.g., COVID-19-related diseases or sectors not related to resource supply, wider developmental impacts or sociopolitical risks). Besides, the final dataset (28 studies), which was closely studied, resulted in several publications indicating similar country-level insights (e.g., on increased food insecurity). Therefore, and considering the limited scope of this paper, we used representative papers for a common argument, and later added the ones with complementary insights. The paper’s analysis of COVID-19’s developmental perils is placed within a growing body of literature, and we therefore focused on key sectors such as health (as the baseline impact), water, food and security. Education, water security and climate change also feature in this analysis in relation to compounded impacts on (resource supply) security, e.g., educational disruptions or climate-related events causing food impasses and security concerns. Future studies can relate to other impacts or seek to quantify them across the Sahel.

## Results: assessing and outlining the distinctive COVID-19 case of the Sahel

### Health sector: pandemics mitigation and aid bottlenecks

In fighting COVID-19 pandemic, the Sahel countries adopted several containment and mitigation measures recommended by WHO, e.g., quarantines, confinements, travel restrictions and closing of education institutions and public places. Mandating masks in public places has become a main action to reduce the spread of COVID-19 in Burkina Faso, Mauritania, Mali, Senegal, Nigeria and Chad (OECD, 2020b). Imposing of COVID-19 containment measures in the Sahel has been very difficult as authorities complained about citizens’ non-compliance with directives and recommendations. This attitude results from the fact that a large part of the population in this region is comprised of irregular workers who can lose their income if they stay home. Besides, access to COVID-19 testing has been limited in the region. Evidence shows that the majority of population in Sahel and low-income countries have experienced some form of income loss due to COVID-19 (Josephson et al., 2021). Some governments provided cash funds or food supplies received from international organizations or from own resources. The Nigerian government, for instance, created coronavirus intervention fund of $1.39 billion to fight COVID-19 (Africa News, 2020). Although such funds have been intended to help vulnerable groups abide by the lockdown, they have been limited and only covered the need of a small part of the population.

**Fig. 5:**
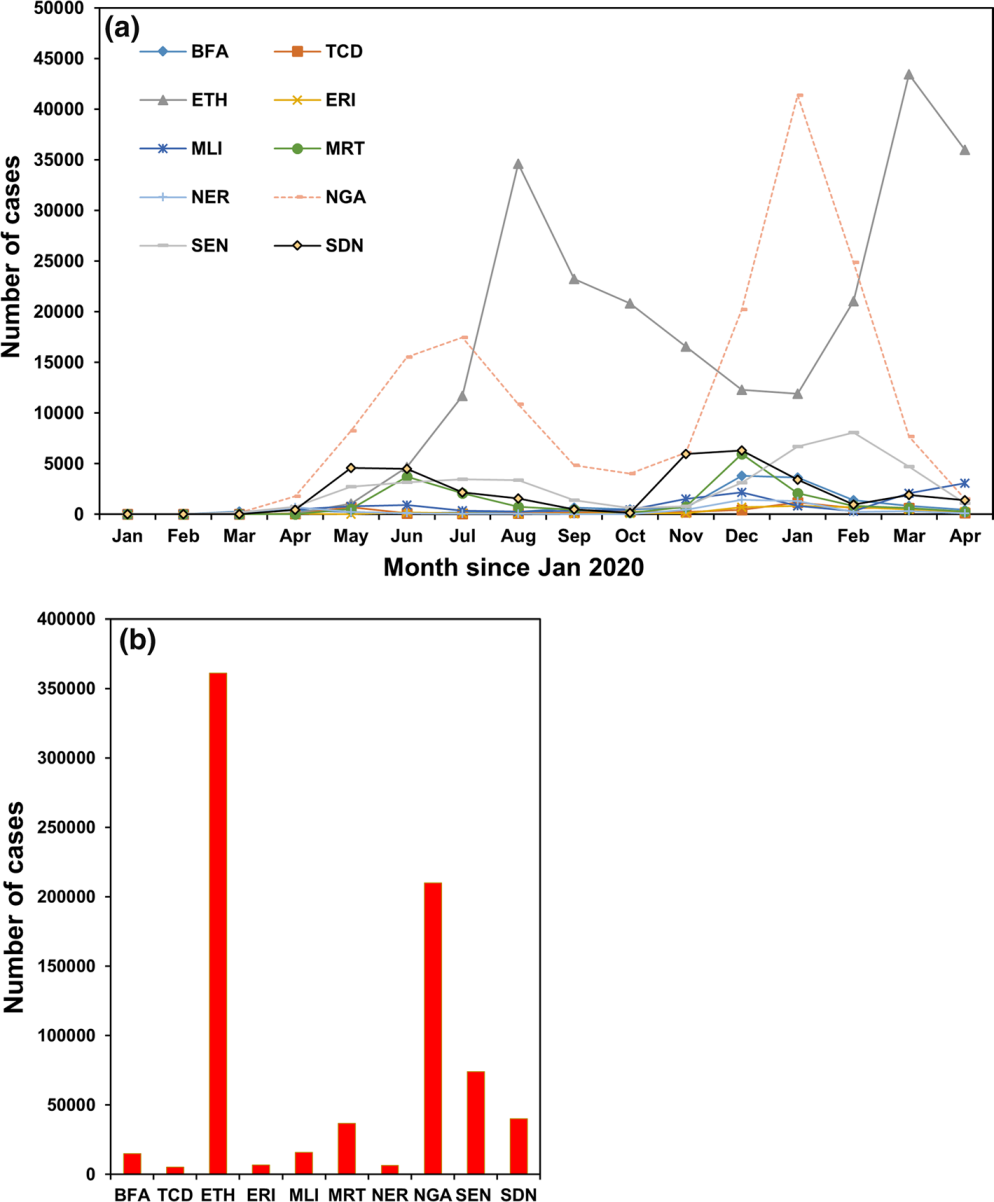
Number of cases of COVID-19 in the Sahel countries. **a** Monthly and **b** total till October 2021. Data source: WHO (2020a). See Fig. 1 or Table 1 for abbreviations

With regard to the progression of the pandemic, COVID-19 cases increased rapidly, reached a peak in July, August and September 2020 in most of the Sahel countries (Fig. 5a). As of October 2021, the total number of cases were spectacularly varying in the region with confirmed cases exceeded 700 thousands (Fig. 5b) compared to 6 million in the whole Africa, and with an average mortality rate of 2% that is similar to the global average. The highest number was recorded in Ethiopia (> 360,000 cases and 6316 deaths) and the lowest was in Chad (5000 cases and 174 deaths) according to WHO (2021a). However, these figures might still not be the real figures due to the weak national healthcare infrastructures in detecting all cases and also due to many people abandoning reporting in fear of being stigmatized by their infection. In fact, COVID-19 has amplified problems of the fragile health care systems in Africa—a continent that has been found unprepared to deal with this pandemic (Elebesunu et al., 2021).

The ability of the Sahel countries to mitigate COVID-19 impacts has also been relying on international aid; however, the aid commitments and delivery have faced some bottlenecks. After declaring COVID-19 as a pandemic, the WHO called for a COVID-19 Solidarity Response Fund aiming at providing funding from action partners of at least US$ 675 million to effect crucial responses in most vulnerable countries. Until November 9, 2020, only around US$ 250 million was collected. The solidarity fund was created at the request of WHO by the United Nations Foundation in Partnership jointly with the Swiss Philanthropy Foundation. In April 2020, the first United Nations “Solidarity Flight” left Addis Ababa, Ethiopia, in order to transport vital medical cargo to all countries in Africa and to contain the spread of COVID-19 (WHO, 2021b). In June 2020, the UN refugee agency (UNHCR) appealed for urgent $186 million to facilitate its humanitarian assistance intended to prevent the acceleration of existing conflicts in the Sahel region as a result of COVID-19 (UN News, 2020a). In 2021, the WHO launched an appeal for US$ 1.96 billion to fulfil the requirements of the 2021 Strategic Preparedness and Response plan (WHO, 2021b). Funding needs are likely to increase as this outbreak evolves.

It is quite difficult to track funding flows in the Sahel region due to lack of data and/or discrepancy between funding pledges and reality. As shown in Table 2, the main funding parties and aid programs for the region are the World Bank group (WB), International Monetary Fund (IMF) and World Food Programme (WFP). Other partners also financially supported some countries in the region through the United Nations International Children’s Fund (UNICEF, 2021), e.g., funding from the UK (£19 million) and from the European Union (€10 million) to Ethiopia and lifesaving supplies to Chad (UNICEF, 2020a). In October 2020, the UN Secretary-General appealed for $2.4 Billion to cover humanitarian needs in the Sahel region through the next year, warning that the region is at ‘a breaking point’ (United Nations, 2020a). In fact, COVID-19 has aggravated the health and humanitarian situation in the region while some of the newly rising pressures are transboundary. In November 2020 for example, Sudan received more than 40,000 Ethiopian refugees who fled across the border to eastern Sudan following the eruption of conflict in the Tigray region in northern Ethiopia, and the influx was expected to increase to 100,000 refugees over the next six months due to continued fighting (OCHA, 2020b). The Sudanese health officials called for health aid to the refugees in the refugee camps against COVID-19, AIDS, hepatitis, tuberculosis and other illnesses (Sudan Tribune, 2020).

**Table 2:** COVID-19 response funding to the Sahel region

Funds and aid programs (April 2020 till April 2021)	BFA	TCD	ETH	ERI	MLI	MRT	NER	NGA	SEN	SDN
WB: World Bank Group (funds in millions of US$)^1^	21.15	16.9	347.5	0	25.8	75.2	13.9	0	20	0
WB: World Bank Group (Boosting funds in millions of US$)^2^	800	15	0	0	0	0	0	0	0	0
IMF: International Monetary Fund (funds in millions of US$)^3^	115.3	183	411	0	$200	130	114.5	0	442	0
WFP: World Food Programme^4^	Yes	Yes	Yes	Yes	Yes	Yes	Yes	Yes	Yes	Yes
OIC: Organization of Islamic Cooperation (OIC)^5^	N/A	N/A	N/A	N/A	N/A	Yes	N/A	N/A	Yes	N/A
MSF: Médecins Sans Frontières (patient treatment)^6^	N/A	N/A	N/A	N/A	Yes	N/A	Yes	N/A	N/A	N/A
United Arab Emirates (tons of healthcare supplies)	8	N/A	33	N/A	6	18	6	N/A	N/A	11

^1^In March 2020, the World Bank announced the COVID-19 Fast Track Facility to support countries’ response to the pandemic. It addresses both emergency containment and mitigation needs for COVID-19, including strengthening countries’ health systems to treat severe cases and save lives. Source: https://www.worldbank.org/en/news/factsheet/2020/06/02/world-banks-response-to-covid-19-coronavirus-in-africa

^2^On December 15, 2020—The World Bank approved three projects to support the economic recovery and improve access to and the quality of basic social services in Burkina Faso and Chad. These projects will help both countries respond to the impact of the COVID-19 pandemic and the humanitarian crisis. The World Bank also confirmed Burkina Faso’s eligibility for the Prevention and Resilience Allocation. (PRA)

Source: The World Bank Boosts Support to the Sahel for a Resilient Recovery from the Security and Economic Crisis

^3^IMF granted Emergency Financing and Debt Service Relief for Sub-Saharan Africa in total amount of $14,597 Million or 32 Countries in this region. Source: https://www.imf.org/en/Topics/imf-and-covid19

^4^In order to respond to COVID-19, WFP has ramped up support to fight against hunger by giving food assistance and cash money in some areas to support poor families. WFP provided support to other organizations through expert knowledge (e.g., in data collection and analysis, policy and advocacy support) and logistics capacity in order to insure the reach of humanitarian aid and health supplies to the target areas. Source: https://www.wfp.org/emergencies/covid-19-pandemic

^5^The OIC through the Islamic Development Bank Group (IsDB) has launched a $2.3 billion Strategic Preparedness and Response Programme to help cushion the adverse health, social and economic effects of the novel coronavirus (COVID-19) pandemic in the OIC Member States. Source: https://anba.com.br/en/mauritania-gets-financial-grant-to-fight-coronavirus/

^6^The COVID-19 response of MSF focuses on three main priorities: supporting authorities to provide care for COVID-19 patients; protecting people who are vulnerable and at risk; and keeping essential medical services running. Source: https://www.msf.org/covid-19

*BFA* Burkina Faso; *TCD* Chad; *ETH* Ethiopia; *ERI* Eritrea; *MLI* Mali; *MRT* Mauritania; *NER* Niger; *NGA* Nigeria; *SEN* Senegal; *SDN* Sudan; *N/A* not-applicable or non-available

Additionally, there existed several bottlenecks with regard the COVID-19 vaccine. As a new disease, COVID-19 had no ready drug, but the only possible control of this pandemic was through the prescribed precaution measures and later through immunization. With several vaccines deployed in late 2020 and early 2021, rich countries have already vaccinated millions of their population. Arrival and administration of vaccines to poor countries, including the Sahel region, show poor performance. Until November 2021, only 42 million doses of different approved types of vaccines were received in all Sahel countries with average administration percentage of 33% (WHO Regional Office for Africa, 2021). The very limited capacity of manufacturing the vaccine locally in the Sahel is compounded by financial and logistic constraints as well as a lack of adequate supply chains of vaccine doses. For example, difficulties might arise with regard to maintaining a cold chain for a proper distribution of the vaccines, especially to rural areas. This situation resembles that of the HIV drugs as Africa has been lagging years behind the world in controlling the spread of diseases (Nkengasong et al., 2020). Meanwhile, the WHO and the United Nations Children’s Fund (UNICEF) had submitted the Vaccine Readiness Assessment Tool to all 47 African countries with a roadmap to plan for the introduction of COVID 19 vaccines (WHO, 2020). In summary, the long-standing vulnerabilities and negative impacts related to fragile health systems, a sudden COVID-19 outbreak and a lack of access to the vaccines and therapeutics have created fertile ground for regional instability, which has worsen the resource supply security and safety as to be discussed in next chapters.

### Water and food: climate risk, conflicts and resource supply security

Over the course of the pandemic, sub-Saharan countries ranking low on food security performance (e.g., suffering from malnutrition) had also witnessed increased rates of fatal COVID-19 cases (Mertens & Peñalvo, 2021). The pillars of attaining food security are availability, accessibility, utilization and stability (FAO, 2006), and recently extended to agency and sustainability (HLPE, 2020). The agricultural systems, particularly the rainfed, in the Sahel region are notorious for erratic rainfall conditions, drought risks, aridity and land degradation (Elagib, 2014; Elagib et al., 2019, 2020) and desert locust outbreaks. Driven by hydro-climatic constraints and fueled by population growth, a risk spiral increases the need for water security for domestic, agricultural and industrial use, thus increasing the stress on the water availability (Falkenmark, 1989). On the one hand, it is widely accepted that the water scarcity in the region is economic in nature, but on the other hand, mismanagement aggravates physical scarcity and jeopardizes the access to clean and adequate water supply (Naik, 2017). Water security in the region does not only concern health, agricultural, educational and economic development, but also relates to peacemaking and political stability (Graves et al., 2019). Water-, food- and climate-related risks will interact with the pandemic-related issues, and can ultimately cause a rise in water and food insecurities as well as malnutrition.

The Sahel is a region of developing and under-developed countries with the above-mentioned constraints inherent in their systems. Therefore, the likely long-lasting COVID-19-related impacts will present challenges related to the increasing demand for water in all the sectors and to the accessibility to safe water quality even for most basic needs, such as drinking and sanitation (Sivakumar, 2020). Through food insecurity and inadequate nutrition of people in extreme poverty, the impacts could be long lasting (Laborde et al., 2020). Just before the pandemic has stricken the region, a desert locust outbreak occurred across the eastern part of the Sahel, plagued the agro-pastoral areas and posed a threat to crop, food security and livelihoods on already vulnerable communities (Salih et al., 2020). In the pastoral context, for example, Griffith et al. (2020) enumerated a number of COVID-19-related impacts, namely direct mortality and morbidity, constrained grazing mobility and restricted access to pastoral areas and markets. Griffith et al. (2020) argued that movement restrictions as a measure of limiting the spread of COVID-19 would restrain the desert locust control. COVID-19 and associated lockdown measures are likely to affect international and local rice trade disruptions in the western part of the Sahel in the short, medium and long terms (Arouna et al., 2020). Arouna et al. (2020) proposed several categories of impacts of COVID-19 on fundamental value chain operations: access to agricultural inputs; procurement of paddy for traditional and upgraded mills; logistics; financing rice growing; a trade-off between increasing the human resources productivity and efficiency and the application of social distancing in operating the mills; impacts on marketing and sales due to massive loss of jobs. Without appropriate actions, switching crop types by some farmers or completely abandoning farming by young farmers are expected consequences (Ayanlade & Radeny, 2020).

Stemming from the lockdown measures and the associated effects in terms of a rise in food prices, an increase in unemployment, a decrease in nominal households’ incomes and a decrease in remittances in Burkina Faso, several scenarios and ramifications for food security are highlighted, viz., widening deficit of food for poor households and drop in the consumption of food, fruits, vegetables, meat and fish below the drought control standard (Zidouemba et al., 2020). Research on sub-Saharan Africa showed the most vulnerable (e.g., women, the poor and the uneducated) to be suffering most from COVID-19-related food insecurities in Sub-Sahara Africa (Dasgupta & Robinson, 2021). In 2020, Sudan suffered from a complex humanitarian crisis characterized by political instability and protracted civil conflicts leading to 1.8 million internally displaced people, sharp economic decline with an inflation rate increased by 214%, unprecedented severe flood affecting more than 875,000 people associated with disease outbreaks, endemic diseases and 1.1 million refugees hosted from South Sudan, all of which resulting in an estimated 9.6 million severely food-insecure people (ACAPS, 2020; IPC, 2020). As the country’s disability to import vital commodities such as wheat escalated, people queuing in front of bakeries for hours to buy bread had become a common scene in Sudan, a situation that called some countries to assist in, and the WFP to facilitate delivery of, wheat relief (OCHA, 2020a). Containment measures were re-introduced later due to rising number of confirmed COVID-19, thereby aggravating this humanitarian situation (ACAPS, 2020). On top of this critical situation, and upon the conflict that is taking place in the Tigray region in Northern Ethiopia, comes the arrival of the large number of Ethiopian refugees to the Sudanese territory in the eastern region. The UN Refugee Agency has launched an emergency relief plan to provide the refuges with basic services, including shelters, water and food supply (UNHCR, 2020). The agency also appealed for urgent access to the needy civilian, Eritrean refugees in the Tigray region in fear of running out of food supplies (UNHCR, 2020). These complexities arise synchronized with a politically-sensitive issue of water resources availability and management in the Eastern Nile Basin related to the filling of the Grand Ethiopian Renaissance Dam (GERD), involving Ethiopia and Sudan in the Sahel and Egypt (Basheer et al., 2020). The GERD is also expected to pose negative environmental impacts in Sudan and Ethiopia, implying risks to the agricultural activities, food security, ecosystems and health despite some intended benefits to both countries (Elagib & Basheer, 2021).

### Security-related concerns: public order, stability and terrorism

Over the course of the COVID-19 pandemic, several concerns have been expressed with regard to potential spillovers on peace and security in the Sahel region. In early May 2020, the UN Secretary-General warned of Jihadist groups in the Sahel exploiting pandemic to increase attacks (France24, 2020). These terrorist groups have in fact actively sought to exploit the overburdened security forces during the pandemic by increasing attacks (Coleman, 2020). So far, there has been an upsurge of terrorist activity in the Sahel and West African regions in 2020, with increased attacks reported in Mali, Burkina Faso and Nigeria (Kishor, 2020; Mednick, 2020). On the one hand, it might be too early to establish a direct link to the COVID-19 pandemic. On the other hand, COVID-19 seems to accelerate the trend of escalated violence which started well before the pandemic, resulting in 1463 armed clashes and 4623 civilians killed from 2012 to 2019 with the highest uptick in violence reported in 2019 (Raleigh et al., 2020). Despite the associated mobility restrictions, the COVID-19 crisis provides a strategic opportunity for terrorist groups to step up attacks, increase propaganda and recruitment against national governments and utilize emerging technologies (Basit, 2020; Norlen, 2020; Raleigh et al., 2020). COVID-19 can create an environment susceptible to exploitation by terrorist groups, e.g., anti-government sentiments, increased radicalization, emergence of new forms of attacks (bio- or cyber-terrorism) or cuts in funds for international security cooperation (Ackerman & Peterson, 2020).

Since the pandemic is already emerging as an accelerator of instability, the consequences of COVID-19 on security and public order in the Sahel will become more evident in the next couple of years. For example, the pandemic has caused economic devastation in Mali, while the parliamentary elections were held in March 2020 under severe restrictions and, thus, a low turnout of 24%. These factors might have had speeded up the coup d’etat against President Keïta in August 2020, as one of the first overthrown governments in the coronavirus era (Taylor, 2020). In May 2021, another coup d’etat took place. Burkina Faso has now become one of the most unstable countries in the region due to reasons such as insurgency, abuses by security forces, floods and economic hardship caused by COVID-19 (International Committee of the Red Cross (ICRC), 2020; Turse, 2020). Increased violence across the region and the COVID-19 crisis have resulted in around 12 million children in Burkina Fasco, Mali and Niger temporarily (up to four months) out of school, and 776,000 missing the entire year, with some schools making place for displaced people from violence (Norwegian Refugee Council, 2020). In January 2022, a successful coup d’etat was launched in Burkina Faso following some attempts in 2021. In Nigeria, and despite lockdown measures, a significant increase in crimes has been recorded in comparison with pre-COVID-19 situation (Okolie-Osemene, 2021). COVID-19 has also resulted in grievances against national governments that were seen as restricting civil liberties during the pandemics and losing control over (peripheral) state territories (Bisson et al., 2020). Political distrust to the government has also undermined compliance to COVID-19 in Nigeria, thus facilitating the spread of the virus (Ezeibe et al., 2020). Overall, the COVID-19 pandemic has increased instabilities, aggravated the development and supply insecurities and slowed down political transitions, e.g., in countries such as Ethiopia or Sudan (Verjee, 2021). Elections were conducted in 2020 in Niger and Burkina Fasco despite COVID-19. Sudan’s political turmoil continued with no roadmap agreed on until now—March 2022. Ethiopia postponed its 2020 national elections until 2021 due the pandemic. This postponement was held, at least partly, as one trigger for the ongoing conflict in the Tigray Region, causing serious concerns about food security in this region. Ethiopia has also suffered from the consequences of returned labor migrants from the Middle East (up to 25,000 migrants until mid-August 2020), thus jeopardizing livelihoods of some population groups (Murzakulova et al., 2021).

## Discussion

### COVID-19’s long-term implications: anticipating a game changer for development

The progression of the COVID-19 crisis in the Sahel region elicits the accumulating vulnerabilities of the recent decades, e.g., growing populations and associated poverty or food insecurities, increasing climate variability as well as deteriorating security. Together with the direct health- and economic-related impacts of the pandemic, a perfect storm of humanitarian and economic perils is ensuing. In this context, we highlight in this section some important implications for the Sahel region from the compounded effects of resource supply insecurities, economic jeopardy and increased political instability in the aftermath of the COVID-19 crisis.

First, it has become evident that the COVID-19 crisis derails the development agenda in the Sahel on important parts such as poverty reduction, food security, health and well-being. This is not surprising since the COVID-19 crisis is expected to affect the long-term outlook for achieving key parts of the global development agenda such as the Sustainable Development Goals (SDGs), both in the Sahel and globally (Barbier & Burgess, 2020; Sachs et al., 2020). The Sahel region has had some tangible improvements with regard to the SDGs (see Sect. 2), which can be wiped out in the next years. For the Sahel countries to return to track, a great effort and funds are needed. The pandemic’s indirect effects stemming from strained health systems, household income loss, and disruptions to care-seeking and preventative interventions, such as vaccination, may be substantial and widespread. Other sectors, such as education, are hit hard, thus aggravating the earlier mentioned supply insecurities since school feeding is an important service in Sahel countries (Abay et al., 2021).

Second, the COVID-19 pandemic clearly shows that health sector outcomes are linked to food security performance. This conclusion might be only applicable to vulnerable and low-income countries such as the Sahel countries. In this paper, we have argued that the weak health systems in the Sahel and disturbances of health services during the COVID-19 crisis have affected food security. At the same time, evidence shows that malnutrition and lack of food access have aggravated the health crisis (Mertens & Peñalvo, 2021). Therefore, more efforts and rigid preliminary healthcare plans are important to save citizens’ life, especially the vulnerable groups like children, pregnant women, old people and patients with chronic diseases (Dasgupta & Robinson, 2021; Elebesunu et al., 2021). COVID-19 has also shown that these efforts need to incorporate sustainable and more comprehensive approaches such as One Health strategies addressing multi-sectoral and multidisciplinary health issues, i.e., linking human health to environmental and animal healths (Ayobami et al., 2021).

Third and finally, the Sahel case reiterates the need for comprehensive approaches toward food security to stand against sudden disturbances. While such approaches have to incorporate access to food, political stability and long-term resilience of supply, crisis response through (international) cooperation has emerged as an essential element of these approaches. For example, on the regional scale, governments are required to prepare cooperated and coordinated post-pandemic food crisis emergency responses. Recovery plans are indispensable region-wide through UN agencies while on the country scale, farmers need to adjust the growing calendar by integrating science (early warning systems, seasonal forecasting and crop science) and indigenous knowledge (Ayanlade & Radeny, 2020). Improved synergies, coordination and utilization of resources within (food) aid agencies is needed while aid agencies focus on emergency supply toward food security. International aid will remain important, particularly for disaster-prone areas. While large international aid would not ease water scarcity in the region without effective governments for better democracy and governance (Naik, 2017), short-, medium- and long-term policy options need be designed to help governments of the Sahel countries mitigate the COVID-19 impacts on food security (Arouna et al., 2020).

### Lessons learned? Way forward for a public sector led resilience

The COVID-19 pandemic has aggravated underlying vulnerabilities in the Sahel with regard to development, supply security and weak public sectors. On the short term, responses to COVID-19 had to prioritize action that seeks to avoid a worst-case scenario of a highly aggravated crisis. To ensure long-term resilience and adequate responses to future shocks, the analysis in this paper highlighted some specific lessons that emphasize the role of public leadership. These lessons are summarized in the following:

- *Strengthening response capacities through state-based cooperation:* COVID-19 mitigation efforts highlighted the importance of building alliances (e.g., through African Union or UN) to improve access to vital COVID-19 supplies. Therefore, the Sahel countries need to assess joint positions and interstate cooperation toward improving their access to vital supplies during crises, e.g., food, therapeutics and vaccines. The region got most of its vaccine doses through the COVID-19 Vaccines Global Access (COVAX) initiative, backed by the World Health Organization, the European Commission, Gavi, the Vaccine Alliance, alongside key delivery partner UNICEF (WHO, 2021c).
- *Rethinking long-term food security and the role of international aid:* The pandemic has shown the importance of investing in national disaster relief programs and empowering the associated institutions to ensure access to basic supplies. Within this notion, the reliance on international humanitarian aid in crisis might not be sustainable since COVID-19 has raised concerns about self-interested aid distribution and ability of rich countries to finance their aid commitments (Brown, 2021; Kobayashi et al., 2021). With regard to food security, one of the main insights from the COVID-19 crisis is that a long-term reorientation toward sustainable local agriculture is worth considering (Al-Saidi & Hussein, 2021). Alongside sustainable local agriculture, and in order to ensure a smooth operation of agricultural input markets and food supply chain, there are many necessary public support measures during the COVID-19 crisis such as social safety programs, accessible household loans, extensions of payment deadlines and tax exemptions for food companies (Ayanlade & Radeny, 2020; Griffith et al., 2020; Laborde et al., 2020; Zidouemba et al., 2020). Households in the agro-pastoral sector who adhere to public health measures related to COVID-19 should be prioritized to food, income and nutrition security (Griffith et al., 2020).
- *Improving monitoring, communication and public sector performance*: Exchanging information, improving awareness and transparency can increase legitimacy of action by governments/donors and hinder exploitation. Ramping up pro-poor programs and social safety nets for the most vulnerable is highly relevant during the pandemic, with a focus on peripheral areas and marginalized groups (Zidouemba et al., 2020). COVID-19 can be seen as an opportunity for improving public sector performance in general, but those agencies related to risk management and disaster relief in particular. Such effort can pre-empt security deteriorations or the exploitation of vulnerable population groups by non-state and violent actors.
- *Investing in peace and public order during crisis:* We have highlighted in this paper how security concerns have been important ancillary impact in the Sahel, thus enlarging supply problems and sociopolitical risks. During crises, and as long-term priorities, peace and political reconciliation are probably the most urgent concerns. The African Union’s declaration for 2020 as the year for “Silencing the Guns” came at a very difficult time for the region. Peace is key to save people, open roads for humanitarian aids and encourage large groups of farmers and workers to revert to production.

## Conclusions

The COVID-19 pandemic was an unwelcome pressure resulting in multi-facetted impacts for health, economic and environmental securities for the already fragile developmental context of the Sahel region. This paper contributes to the scarce literature using systematic analyses of the compounded effects of the pandemic at hand. Besides, the consequences of the Sahel COVID-19 case are poorly understood in terms of the interplay of impacts and their ramifications on resource supply security. We therefore contextualized this case by highlighting environmental and sociopolitical aggravators, and by presenting evidence on the progression of the specific Sahel scenario. As the Sahel region is largely underprepared and recently more vulnerable, the COVID-19 presents a perfect storm of humanitarian jeopardy due to the cross-sectoral nature of this impactful pressure. Moreover, this paper contextualized the COVID-19 impacts within the underlying vulnerabilities in the Sahel and the bad timing of this event. The economic growth has slowed down in the last decade while the humanitarian crisis has worsened due to environmental degradation, natural disasters and security-related challenges. Food insecurity has become an even higher concern in the time before the pandemic due to climate variability, disasters such as locust swarms or floods, and migration pressures. The Sahel region is prone to disasters, but it has so far been an underperformer in global indices that measure food, health, energy and political securities. International aid has been crucial for increasing readiness and improving responses for pressures such as COVID-19.

We have shown in this paper how the COVID-19 trajectory in the Sahel region has been quite specific, with several ancillary effects have led to an enlarged sociopolitical risk. Such effects include unequal access to therapeutics and vaccines, discriminatory mobility restrictions, aid bottlenecks, displacements and security-related disruptions. We presented evidence for demarcating the unique Sahel scenario and provided examples from the sectors of health, food and basic supply as well as security and public order. With regard to health, we found out that the COVID-19 mitigation efforts have become prevalently reliant on international aid. We tracked some of the funding flows for COVID-19 mitigation, and identified some bottlenecks and constrains for health and vaccine-related aid flow. These constraints include delays in delivering committed aid, providing vaccines and logistics in addition to supply chain limitations. On the scale of food security, COVID-19 has already resulted in several food production disruptions or price effects, and has come after or synchronized with the recent increase of climate-related disasters and of the needs for disaster relief. During the COVID-19 progression, additional pressures of livelihood loss and displacements have materialized due to, for example, severe floods in Sudan or violence in Ethiopia. In this sense, food security was found out to be closely interlinked with health sector outcomes in the Sahel. Finally, in the security realm, increased tensions have been witnessed in the form of exploitation by terrorist groups, rise of anti-government sentiments, delays of elections and restrictions of civil liberties. Such an instability has aggravated the resource supply security in the Sahel.

In the long term, the impacts of the COVID-19 crisis are expected to aggravate underlying vulnerabilities of the Sahel in terms of developmental shortcomings, security concerns and accumulating climatic risks. In this regard, this paper provided a valuable regional view attesting to COVID-19’s potential to derail important parts of the development agenda in low-income countries. It also showed that close interlinks exist between health sector performance, public order and food security, and therefore called for more comprehensive approaches to health (e.g., linking human health to environmental or other health issues) and to food (e.g., more long-term views incorporating local sustainable agriculture). In the Sahel, COVID-19 relating responses sought to avert a worst-case scenario of widespread humanitarian and economic perils. Some lessons from this pandemic include the value of public sector-led responses, such as increasing regional and state-based cooperation, strengthening of healthcare capacities, prioritizing food access, securing and facilitating international aid (while not relying on aid and reinforcing national relief programs), and improving pandemic communication. These responses can mitigate the additional impacts of COVID-19 on the Sahel, but the baseline impacts related to economic difficulties and a temporary setback of the key parts of the development agenda in the region might remain for years to come. Here, this paper did not examine single local disruptions but rather the compounded impacts (with resource supply, particularly food, as a main focus) of the first years of the pandemic in the Sahel. Future research can therefore examine action to tackle long-term impacts as, for example, efforts to enhancing sustainable production and self-sufficiency or rethinking and enhancing resilience plans and programs. Ultimately, the success of the recovery from COVID-19 will vary from one state to another, depending on larger issues such as the capacity to reorganize and readapt development policies, as well as the will to prioritize peace and political reconciliation in a post-COVID-19 world.

## Data Availability

Figure 3 data that support the findings of this study have been deposited in the World Bank Data repository (data.worldbank.com) and African Development Bank countries data (https://www.afdb.org/en/countries). The source of Fig. 4 data is World Health Organization (WHO). Coronavirus (COVID-19) Dashboard. Geneva, Switzerland. https://covid19.who.int. Sources of data in Tables 1 and 2 are indicated in the footnotes. Data supporting discussion and analysis in different parts of the paper are taken from: World Health Organization Data Platform (World Health Data Platform—WHO: https://www.who.int/data#reports); UNICEF Data: UNICEF DATA—Child Statistics (https://data.unicef.org/); UNESCO Institute for Statistics: Data for Sustainable Development Goals: UIS Statistics (http://data.uis.unesco.org/); ReliefWeb (https://reliefweb.int).
